# Nurses and the League of Nations

**Published:** 1919-05-10

**Authors:** 


### THE MATRONS' AND SISTERS' DEPARTMENT.

### NURSES AND THE LEAGUE OF NATIONS.

When the historian of the far future turns to sum up in a few paragraphs the events of a century, it may surely be foretold that among the epoch-making deeds which will move his pen are those of the last days of April 1919. The moment is so solemn, its import so vast and limitless, that it seems well-nigh incredible that we under whose eyes these things have been planned and carried through can have room for aught else in our minds. The generations to come will fail to realise that there could have been any among us so wanting in imagination as not to concentrate our minds on the League of Nations in its first moments of inception, any who passed it by in the multitude of petty interests, finding "no time" to study its great principles or think out what it means for the world.

We are surely doomed to disappointment if we regard the ratification of this League as the opening of a Utopia which will obviate the necessity for further struggles on behalf of humanity. Its worst enemies are the easy optimists who expect to triumph without conflict. Rightly regarded, the League of Nations is a turning-point in the history of our race. It is a hinge on which the great gates of Janus may by stupendous and united pressure be pushed back and closed for ever. From now on the mighty endeavours of mankind, hitherto directed towards mutual conquest and subjugation, are turned in a new direction—the establishment of liberty and justice, the recognition of mutual rights. It is such a volte face, in principle if not yet wholly in method, as poets, prophets, and philosophers have dreamed of throughout the ages. It has been accomplished under pressure of the universal conviction that the old system of aggression and violence has been worked to its utmost limits and broken down. The knowledge that the League owes its inspiration mainly to the English-speaking peoples of the globe should make us very humble, very determined to lend ourselves body and spirit to its principles, very stern to root out from ourselves and from others any impulses towards greed, impatience, or cowardice which might retard its perfect work.

To nurses gathering their energies for the reconstruction of their profession the League of Nations brings a trumpet call to action. Who can doubt that the united forces of the International Red Cross Society will be at the disposition of the League? The time has come for a perception of the truth that no nation can stand or fall alone, and that all are inextricably linked together for good or evil. Great Britain since the conquest of the air is no longer insular. The United States are no longer American pure and simple. We are all European, and Europe can no longer be considered an agglomerate of hostile states at liberty to conquer and re-conquer as opportunity serves. Medical science has shown us the tremendous danger which lurks in trifling epidemics springing into activity in obscure localities, and is equipping men and women in thousands for the task of subduing disease, no longer locally but universally.

An immense quickening of the heart and intellect results from this new citizenship of the world. The days of self-seeking adventure are over, but the adventurous are needed more than ever. Under a reign of peace, with united transport services, with a co-ordinated medical and nursing service for the globe, there can be no limits to the enterprises called forth by the new spirit of progress. Already the International Red Cross Society is welding together a band of nurses and other trained workers who shall be at their disposition wherever required, for stamping out epidemics, assuaging disasters, and banishing that ignorance which spells disease. Far and near the chain will need to stretch. Every improvement in transport brings the world nearer akin, and we may yet live to reckon the departure of a band of nurses by aeroplane to the seat of some distant earthquake or epidemic as among the ordinary events of busy lives.

Nothing can more surely aid to make the League of Nations to prosper and unite mankind than the mental forces of those whose hearts are set on peace. The strong propulsion of will and desire which forms the essence of that exercise we describe as prayer makes itself felt irrespective of time and place. It gathers like a flood of great waters sweeping selfish egotism before it like rotten barriers to a stream. Let it not be said that this great moment in the history of mankind found us heedless of its import, forgetful of the part it calls us all to play.

